# Effects of Unconscious Processing on Implicit Memory for Fearful Faces

**DOI:** 10.1371/journal.pone.0014641

**Published:** 2011-02-01

**Authors:** Jiongjiong Yang, Xiaohong Xu, Xiaoya Du, Cuntong Shi, Fang Fang

**Affiliations:** Department of Psychology, Peking University, Beijing, People's Republic of China; The University of Western Ontario, Canada

## Abstract

Emotional stimuli can be processed even when participants perceive them without conscious awareness, but the extent to which unconsciously processed emotional stimuli influence implicit memory after short and long delays is not fully understood. We addressed this issue by measuring a subliminal affective priming effect in Experiment 1 and a long-term priming effect in Experiment 2. In Experiment 1, a flashed fearful or neutral face masked by a scrambled face was presented three times, then a target face (either fearful or neutral) was presented and participants were asked to make a fearful/neutral judgment. We found that, relative to a neutral prime face (neutral–fear face), a fearful prime face speeded up participants' reaction to a fearful target (fear–fear face), when they were not aware of the masked prime face. But this response pattern did not apply to the neutral target. In Experiment 2, participants were first presented with a masked faces six times during encoding. Three minutes later, they were asked to make a fearful/neutral judgment for the same face with congruent expression, the same face with incongruent expression or a new face. Participants showed a significant priming effect for the fearful faces but not for the neutral faces, regardless of their awareness of the masked faces during encoding. These results provided evidence that unconsciously processed stimuli could enhance emotional memory after both short and long delays. It indicates that emotion can enhance memory processing whether the stimuli are encoded consciously or unconsciously.

## Introduction

Many studies have shown that emotional stimuli can be processed even when participants perceive them without conscious awareness [1]–[4]. The experimental paradigms used frequently for rendering visual stimuli unconscious include backward masking [5]–[7] and binocular rivalry [8], [9]. In the backward masking paradigm, a stimulus is usually presented for a short duration, and then replaced by a mask to interrupt visual processing related to the stimulus and thus excludes awareness. In addition, information that fails to reach awareness still appears to guide our behavior and affect processing of the stimuli that we are aware of [10]. In some situations, unconsciously processed stimuli could affect behavioral performance more profoundly than stimuli that are consciously processed [11]. However, the extent to which unconsciously processed emotional information influences subsequent memory process is not fully understood.

Memory processes consist of encoding, consolidation, and retrieval phases. Based on whether participants consciously retrieve information, memory could be further divided into explicit and implicit memory [12], whether information is *encoded* consciously or not. Note that in an experimental environment, when participants unconsciously process stimuli, it is difficult for them to explicitly retrieve the stimuli. Therefore, implicit memory tests (e.g., subliminal affective priming, lexical decision) are usually adopted to explore the effect of prior unconscious experience on subsequent behavioral performance [12]. Priming effect, a type of implicit memory, is thus defined as changes in reaction time (RT) or response accuracy for repeated items in comparison with new items [12]–[14]. By using subliminal affective priming paradigms [15]–[21], studies found that subjects responded to a target more quickly and accurately when its valence was the same as a prime than when they were different. For example, unconsciously processed negative faces (vs. neutral faces) were more quickly judged as negative [17]. Judgment of neutral targets is also influenced by the valence of primes. Chinese characters were rated higher in likability when preceded by happy faces for 4 ms than when preceded by angry faces. However, when the faces were presented for 1000 ms, the priming effects diminished [19].

There are several issues that need further investigation. One is that subliminal priming effect may be contaminated by a conscious component [9], [15], [22], [23]. For example, stimulus durations around 30 ms in previous studies (e.g., 33 ms in [7]; 20 ms in [15]) are not short enough to ensure unconscious processing, because some participants were able to detect faces with a 33 ms duration [23]. Therefore, it is necessary to use both objective and subjective criteria to separate participants into aware and unaware groups in subliminal affective priming studies.

The second issue is that previous studies did not elucidate whether the subliminal priming effect was different between emotional and neutral stimuli. For explicit memory, emotional stimuli could be remembered better than neutral stimuli, with higher accuracy and/or faster RTs in cued recall and recognition tests. Emotional contexts also enhance memory of subsequent neutral stimuli compared with neutral contexts [2], [24]–[26]. Note that, in addition to the difference in memory retrieval, these studies differ from those using subliminal affective priming paradigms in that stimuli are encoded consciously and retrieved after a long delay [24]. As for implicit emotional memory, although the subliminal priming effect is robust, it is unclear whether the priming effect of emotional stimuli is different from that of neutral stimuli. This issue is important; if true, it would indicate that emotion enhances memory process, regardless of the stimuli are processed consciously or unconsciously.

The third issue concerns how long the affective priming effect lasts. Studies have shown that significant influence of preceded emotional stimuli only occurred at short stimulus onset asynchronies (SOA) [16]–[18], [27], [28]. Affective priming is largest when the SOA is 0–150 ms, and diminishes when the SOA is longer than 300 ms, because automatic activation occurs at a very early stage of information processing, and dissipates quickly [16], [18]. Only a few studies have explored the long-term influence of unconsciously processed emotional stimuli [29]–[31]. For example, when fearful and disgusting pictures were presented for 40 ms, participants were more likely to complete word stems using fearful and disgusting words [30], suggesting that specific emotional responses can be induced without awareness (i.e., participants do not have a conscious emotional experience). Nevertheless, due to possible involvement of conscious processing, it is necessary to address this issue by ensuring that encoding processing is completely unconscious.

In this study, two experiments were designed to investigate whether unconscious processing could enhance implicit emotional memory after short and long delays. To ensure that participants processed faces unconsciously, the backward masking paradigm was used, and subjective and objective assessments for awareness were adopted for each experiment. In Experiment 1, the subliminal affective priming paradigm was adopted. After a masked fearful or neutral face was presented three times, participants were asked to make a fearful/neutral judgment to a target face. Half of the target faces were repeated faces (congruent condition), and the other half were faces with the same identity but a new expression (incongruent condition). In Experiment 2, a long-term priming task was adopted. Participants were presented with a masked faces six times during encoding. Three minutes later, they were asked to make an emotional judgment for congruent and incongruent faces. Note that we presented faces for multiple times during encoding. Studies have suggested that implicit memories are formed after multiple presentations, including unconsciously conditioned reflexes [5], observational learning [32], and implicit learning [33]. Our preliminary data also suggested that priming effect for faces occurs after more than one-time presentation. In addition, because anxiety trait and state influence emotional processing [34], participants were assessed by the State and Trait Anxiety Inventory (STAI) to ensure that they had no obvious change of anxiety status before and after the experiment.

Previous studies have shown that fearful faces could be unconsciously processed, leading to increased activation in the amygdala and subcortical regions [1], [35]. If unconscious activation for fearful faces is sufficient, the priming effect for fearful faces, but not for neutral faces, would be significant after a short delay. In addition, the increased activation in the amygdala could in turn increase attention and cortical activation to fearful faces [2], [26], and could further increase corticosteroid releases [25]. If this also applies to implicit memory of emotional stimuli, the priming effect for fearful faces would occur after a long delay.

## Results

### Experiment 1

Participants were divided into aware and unaware groups by their awareness assessments. The analyses of d′ and A′ confirmed that the two groups of participants had different awareness levels (*F*(1,58) = 31.35 for d′ and *F*(1,58) = 31.22 for A′, both *ps*<.0001). The Prime×Target×Group ANOVA showed a significant interaction among Prime, Target, and Group (*F*(1,57) = 5.65, *p*<.02, partial η^2^ = .09), and a significant effect of Target (*F*(1,57) = 6.74, *p*<.01, partial η^2^ = .11), with other effects did not reach significance (*ps*>.3).

**Figure 1:**
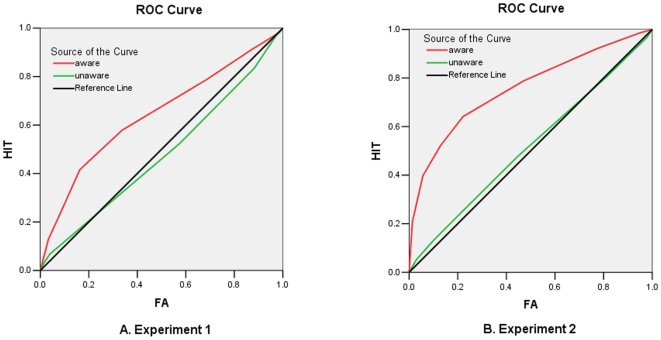
ROC curves for Experiment 1 and 2. The ROC curves were obtained by computing the cumulative Hit and False Alarm rates for each confidence level across participants in unaware and aware groups. The curves for unaware and aware groups were different in both experiments (left for Experiment 1 and right for Experiment 2).

After the sample size was matched between groups, the results also showed a significant difference between the groups for d′ and A′ (both *ps*<.0001). Figure 1 illustrates the ROC curves for both unaware and aware groups. The ROC curves were obtained by computing the cumulative Hit and False Alarm rates for each confidence level across participants in unaware and aware groups. The d′ (vs. 0) and A′ (vs. 0.5) were significantly higher than chance level for the aware group (*t*(17) = 5.24, *p*<.0001 for d′ and *t*(17) = 4.98, *p*<.0001 for A′), but lower than chance level for the unaware group (*t*(19) = 2.27, *p*<.04 for d′ and *t*(19) = 1.74, *p*<.1 for A′) (Table 1). There were no significant effects for accuracy (all *ps*>.1) (Table 2). For RTs, the ANOVA of Condition (congruent vs. incongruent) by group showed a significant interaction (*F*(1,36) = 4.25, *p*<.05, partial η^2^ = .11), with their main effects not significant (*p*>.8). The ANOVA of Prime×Target×Group further showed a significant interaction among Prime, Target, and Group (*F*(1,36) = 4.25, *p*<.05, partial η^2^ = .11), but marginal significance for Target effect (*F*(1,36) = 6.74, *p*<.08, partial η^2^ = .09). There were no significant main effects of Prime (*F*(1,36) = 2.2, *p*>.15), Group (*F*(1,36) = 0.01, *p*>.93), and other interactions (*ps*>.5). After controlling for influence of RT difference in fearful and neutral faces (*F*(1,36) = .21, *p*>.65), the ANOVA for facilitated RTs also showed a significant interaction among Prime, Target and Group (*F*(1,36) = 4.25, *p*<.05, partial η^2^ = .11), with other main effects and interactions not significant (*ps*>.1).

**Table 1:** d′ and A′ scores for Experiment 1 & 2.

	Exp.1		Exp.2	
	d′	A′	d′	A′
Aware	0.65±.52	0.64±.12	1.30±.59	0.78±.08
Unaware	−0.21±.41	0.47±.08	0.12±.30	0.52±.08

**Table 2:** Response accuracy under each condition for Experiment 1 & 2.

	Fear-fear	Neut-fear	Neut-neut	Fear-neut	New fear	New neut
Exp.1	.95±.08	.96±.06	.97±.06	.96±.08	.95±.07	.98±.04
Exp.2	.96±.06	.96±.07	.94±.07	.96±.07	.97±.06	.97±.05

**Figure 2:**
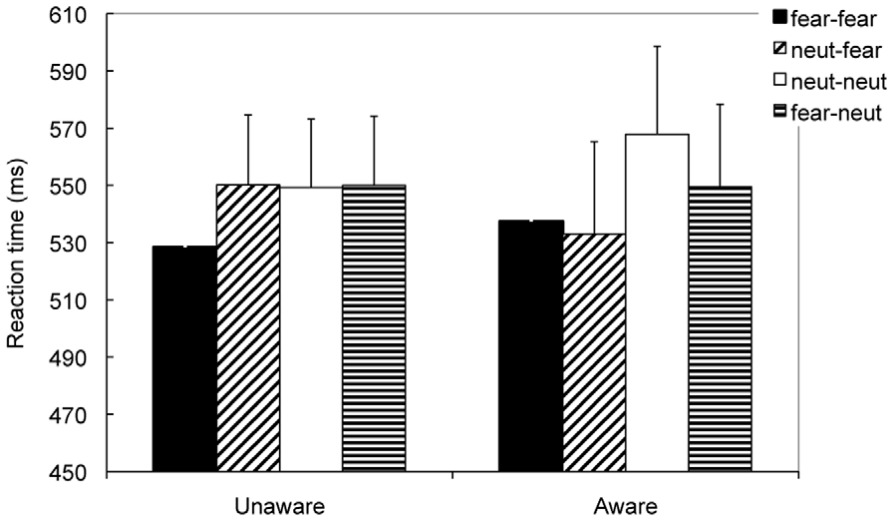
Results of Experiment 1. For the unaware participants, the fear–fear faces were judged more quickly than the neutral–fear faces, but the neutral–neutral faces were judged with RTs comparable to the fear–neutral faces.

The interaction of Prime, Target, and Group was significant, indicating that different priming patterns were obtained for the aware and unaware groups (Figure 2). For the unaware group, fear–fear faces were judged more quickly than neutral–fear faces (*p*<.03), but neutral–neutral faces were judged with RTs comparable with fear–neutral faces (*p*>.9). In contrast, for the aware group, the congruent faces were judged more slowly than incongruent faces, although the simple effects were not significant (*p*>.1).

Experiment 1 showed that only for unaware participants, the fear–fear faces were judged more quickly than the neutral–fear faces, and the neutral–neutral faces were judged with comparable RTs to the fear–neutral faces. Our data was consistent with previous studies [15]–[17], [19], and confirmed that primes could influence subsequent judgments of emotion when participants were unaware of their presentation. The influence may result from the automatic activation of fearful feature of faces in the amygdala when they were unconsciously processed [16], [17]. In contrast, it seems that awareness of the primes hampers the affective priming effect [10], [19], [36], [37].

### Experiment 2

The interval between the prime and target faces was only 500 ms in Experiment 1. It remained to be determined whether the significant priming effect for the fearful faces could occur when the study–test interval is longer. We addressed this issue in Experiment 2 by asking participants to make an emotional judgment 3 min after they were exposed to masked faces for six times. Similarly, participants were separated into unaware and aware groups according to whether they were aware of masked faces by subjective and objective assessments.

The analyses of d′ and A′ confirmed that the two groups of participants had different awareness levels (*F*(1,69) = 111.48 for d′ and *F*(1,69) = 174.51 for A′, both *ps*<.0001). The Prime×Target×Group ANOVA showed a significant interaction between Prime and Target (*F*(1,69) = 4.34, *p*<.05, partial η^2^ = .06), and a significant effect of Target (*F*(1,69) = 15.71, *p*<.0001, partial η^2^ = .19), with other effects did not reach significance (*ps*>.1).

**Figure 3:**
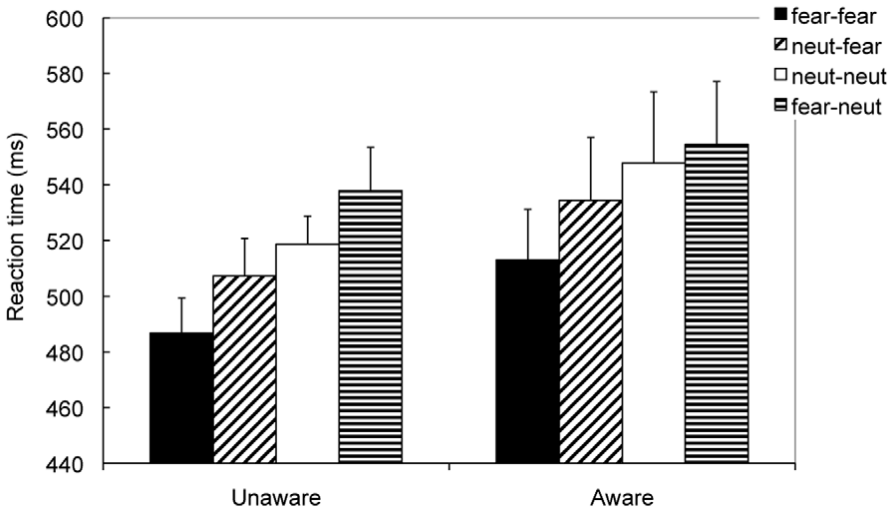
Results of Experiment 2. For both groups of participants, the fear–fear faces were judged more quickly than the neutral–fear faces, but the neutral–neutral faces were judged with RTs comparable to the fear–neutral faces.

After the sample size was matched between groups, the results also showed significant differences between the groups for d′ and A′ (both *ps*<.0001) (Figure 1, right). The d′ (vs. 0) and A′ (vs. 0.5) were significantly higher than chance level for the aware group (*t*(23) = 10.59, *p*<.0001 for d′ and *t*(23) = 17.56, *p*<.0001 for A′), but were comparable to chance level for the unaware group (*t*(22) = 1.19, *p*>.25 for d′ and *t*(22) = 1.03, *p*>.3 for A′) (Table 1). For RTs, the Condition by Group ANOVA showed a significant effect of Condition (*F*(2,90) = 4.49, *p*<.01, partial η^2^ = .10), because congruent faces were judged more quickly than incongruent faces (*p*<.02) and new faces (*p*<.09). The congruent effect was mainly contributed by fearful faces, because the Prime×Target×Group ANOVA showed a significant interaction between Prime×Target (*F*(1,45) = 7.96, *p*<.007, partial η^2^ = .15), with the effects of Prime (*F*(1,45) = 1.21, *p*>.27) and other interactions not significant (all *ps*>.4). The interaction between Prime and Target was manifested as fear–fear faces were judged more quickly than neutral–fear faces (*p*<.002), but neutral–neutral faces were judged at a comparable level of fear–neutral faces (*p*>.2) (Figure 3). Note that both groups showed a similar pattern, with no significant effect of Group (*F*(1,45) = 074, *p*>.4).

Because fearful faces were responded more quickly than neutral faces (519 ms vs. 549 ms, *F*(1,45) = 18.66, *p*<.001, partial η^2^ = .29), we therefore calculated and analyzed the facilitated RTs for each condition. The ANOVA for facilitated RTs also showed a significant interaction between Prime and Target (*F*(1,45) = 7.96, *p*<.01, partial η^2^ = .15), with other main effects and interactions not significant (*p*>.3). The results showed a significant priming effect for fearful faces compared with neutral faces irrespective of the awareness. For accuracy, the aware group had higher accuracy than the unaware group (0.97 vs. 0.95, *F*(1,45) = 4.97, *p*<.03, partial η^2^ = .10), but no other significant effects were found (all *p*s>.10).

Consistent with Experiment 1, the results of Experiment 2 showed that the priming effect was only significant for the fearful faces, whether participants encoded faces consciously or unconsciously. Unlike Experiment 1, participants in the aware group showed a similar pattern to that in the unaware group. It is possible that after six presentations and a 3 min delay, the encoded faces, whether they were consciously perceived or not, were processed in a similar way. These data supported the view that unconscious processing could influence subsequent behavioral performance over time.

## Discussion

The object of this study was to explore the extent to which unconsciously processed emotional stimuli influence subsequent implicit memory after short and long delays. There are two novel findings. First, the significant priming effect for fearful faces was shown using the affective priming paradigm, with the fear–fear faces being judged more quickly than neutral–fear faces for the unaware participants. Second, the significant priming effect for fearful faces was also shown after a long delay using the long-term priming paradigm, whether participants were aware of the primed faces during encoding. These data provided evidence that the affective priming effect existed even when the SOA was longer than 300 ms. In addition, they indicated that emotion enhances memory processes whether the stimuli are processed consciously or unconsciously.

### Unconscious Processing under Backward Masking

It is crucial to ensure that participants process stimuli unconsciously in the subliminal priming paradigm. However, in many previous studies using the backward masking paradigm to render stimuli unconscious, the durations are not short enough to ensure that the processing is unconscious [9], [22], [23]. Thus, controlling participants' awareness level has been a challenge in such studies. In this study, we adopted several approaches to ensure that participants processed the masked faces unconsciously. First, participants were assessed by both subjective and objective criteria. Depending on whether participants were aware of the masked faces, they were divided into two groups. Second, to ensure that the mask did not influence the valence judgment of the target face, the scrambled faces were used as masks. This was different from previous studies that used neutral faces from the same person as masks [5], [22], [23]. Neutral faces are usually judged as untrustworthy and potentially dangerous, and they might lead to activation in the amygdala and visual cortex [38], [39]. In contrast, scrambled faces have advantages of being comparable with faces in visual features (e.g., contour, brightness). They are also meaningless to participants, and have been demonstrated to be effective in preventing conscious processing of stimuli [22]. It should be noted that stimulus duration in the backward masking paradigm was short, thus it would be interesting to use other paradigms (e.g., binocular rivalry) to investigate the implicit emotional memory, although the paradigms may rely on different mechanisms [40].

### Priming Effects for Fearful Faces after a Short Delay

Using the subliminal affective priming paradigm, we obtained a significant priming effect for the fearful faces, but not for the neutral faces, showing that fear–fear faces were judged more quickly than neutral–fear faces. Our study not only confirmed the subliminal affective priming effect that was obtained in previous studies [17]–[19], [21], but also extended them in that the affective priming effect existed even when the SOA was longer than 300 ms. The difference in material type and presentation time may account for the discrepancy between ours and previous studies. We used faces as stimuli, rather than words [27] or pictures [28]. Emotional faces may evoke stronger physiological and neural responses than affective scene pictures [41]; thus, it is possible that fearful faces elicit more activation in the brain even when unconsciously processed, especially when they are presented multiple times.

The facilitation in affective priming is thought to depend on automatic spreading activation in brain regions related to stimulus processing [13], [16]. As the amygdala is related to the affective priming effect [42]–[44], it is possible that after multiple presentations of faces, the amygdala and related subcortical regions are activated especially for the fearful faces [5], [9]. The fearful faces could then attract more attention, and their activation could spread automatically to the related network, thus facilitate subsequent valence-congruent judgment of the fearful faces [16].

Note that affective priming was only obtained for unaware participants. Participants who were aware of the primes showed the opposite pattern (i.e., quicker response for incongruent faces than congruent faces), although the difference did not reach significance. It was consistent with previous studies that reported negative priming effect [34]. This usually happens when the prime duration is longer, or the primes are highly attended. One view claims that when participants are aware of the prime, they will adopt strategic resistance to enhanced influence of the prime. Indeed, negative priming effects are larger when participants are aware of primes (vs. unaware), or remember the primes (vs. forget) [19], [37].

### Priming Effects for Fearful Faces after a Long Delay

Previous studies on explicit emotional memory have shown that, with different types of encoding tasks, emotional stimuli are usually better remembered than neutral stimuli [24]. Unlike these studies, we asked participants to process the faces unconsciously, and tested memory with the typical long-term priming paradigm. Our results and others [29], [30], [45] indicated that emotion enhances memory processes, whether the faces are processed consciously or unconsciously.

We found significant priming effects for fearful faces not only after the short delay, but also after the long delay for unaware groups of participants. But we did not find priming effects for neutral faces in both experiments, although the effects showed a non-significant pattern in Experiment 2. The two experiments varied in some method details. For example, participants were asked to make judgments seconds after a face was presented three times in Experiment 1, but were tested three minutes later after all faces were presented six times in Experiment 2. One possibility was that with more learning exposures and longer retention, there is more automatic spreading activation in related regions [15], [16], which led to different results on neutral faces in the two experiments. Another possibility was that there are distinct mechanisms mediating subliminal affective priming and long-term priming effects. To support the second possibility, both aware and unaware groups of participants illustrated similar patterns of priming effect in Experiment 2, but they showed different patterns of priming effects in Experiment 1. In addition, a recent study found that short-lag priming is related to decreased activity in the occipito-temporal cortex, but the long-lag priming effect is also related to decreased activity in the ventral frontal cortex and increased activity in the parietal and frontal regions [46].

On the other hand, there could be a common mechanism underlies the long-term priming effect for implicit and explicit emotional memory. Previous studies have suggested that explicit emotional memory depends on enhanced attention [48] and memory consolidation mechanisms [25]. Unconsciously processed stimuli could enhance the activity in the amygdala. The activation in the amygdala and prefrontal cortex could also increase attention and cortical activation [2], [26], [36], [46], [47]. Cholinergic enhancement can also enhance the priming effect for emotional faces [48]. The longer the retention, the more enhanced memory might be for emotional stimuli [24]. On the other hand, previous studies have found that priming effect could change from positive to negative as a function of prime duration [36], [49]. The extent to which the significant priming effect for fearful faces lasts over time needs further investigation. In addition, fearful faces differed from neutral faces in both the levels of arousal and valence in our study; and there was discrepancy between the race of the subjects (Chinese) and the race of the stimuli (both eastern and western faces). Future studies are needed to dissociate the effects of valence, arousal and race, to determine whether there are different mechanisms for priming effects and explicit memory.

In conclusion, our study found that after participants encoded masked faces but were not aware of them, they judged fear–fear faces more quickly than neutral–fear faces after short and long delays. These data provided evidence that unconsciously learned emotional stimuli facilitate subsequent emotional memory over time. Considering that some emotional disorders, such as anxiety, phobias, and posttraumatic stress disorder, may also be related to multiple unconscious exposures to emotional stimuli, our study indicated how long and to what extent unconscious processing is sufficient for significant priming effects for emotional faces.

## Methods

### Experiment 1

#### *Participants*

Sixty-one (21.87±1.73 years old, 30 male) right-handed, healthy native Chinese-speaking students at Peking University took part in this experiment. They had normal or corrected-to-normal vision, and no history of neurological trauma or psychiatric disorders. All participants were paid for their participation and gave written informed consent in accordance with the procedures and protocols approved by the Human Participants Review Committee of Peking University.

#### *Materials*

Sixty face photographs of 30 persons (15 male and 15 female) showing either fearful or neutral expressions were used. Some of them were selected from the NimStim database, and others were taken for Chinese students. Half of the faces were western people, and half eastern people. The faces were selected based on their valence or arousal scores, determined by a group of 37 Chinese participants at Peking University using a nine-point Likert scale. Their rating scores showed that, the fearful faces were more unpleasant (3.01±.30 vs. 4.49±.39, *t*(59) = 25.12, *p*<.0001), and more arousing (6.23±0.28 vs. 4.20±0.26, *t*(59) = 45.25, *p*<.0001) than the neutral faces. Phase-scrambled images for all faces were created to serve as masks and control stimuli, each preserving color and the spatial frequency of the original picture without depicting face form.

Three factors were included in Experiment 1: Prime (fearful, neutral) and Target (fearful, neutral) as within-subjects factors, and awareness group as a between-subjects factor. In turn, there were four prime–target conditions for each group: fear–fear, fear–neutral, neutral–fear, and neutral–neutral faces. The prime and target faces were the same in the fear–fear and neutral–neutral conditions, so they were also referred to as congruent condition. The neutral–fear and fear–neutral conditions were referred to as incongruent condition. All together, there were 20 affectively congruent (10 fearful–fearful, 10 neutral–neutral), and 20 affectively incongruent (10 fearful–neutral, 10 neutral–fearful) facial stimuli. Another 20 scrambled prime–facial target pairs (10 scrambled prime–fearful targets, 10 scrambled prime–neutral targets) were included to be new fearful and neutral faces.

#### *Procedure*

**Figure 4:**
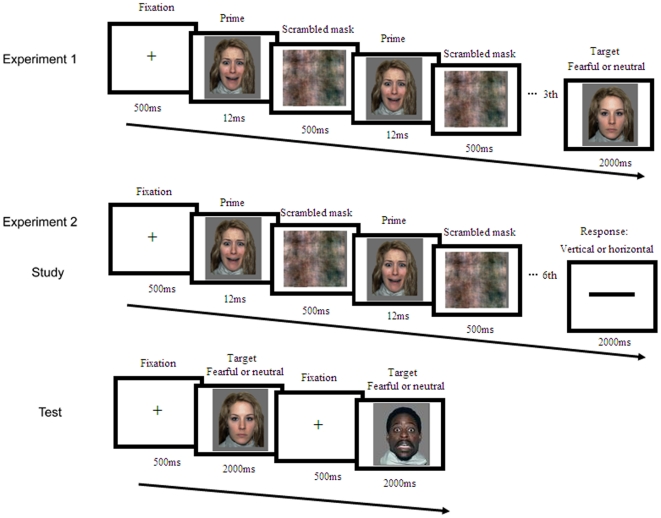
Experimental procedures for Experiment 1 and 2. In Experiment 1, the masked prime faces were presented for three times, and an emotional judgment was performed. In Experiment 2, the masked faces were presented for six times during study, and participants were asked to judge the orientation of the bar. The emotional judgment was performed three minutes after the study phase.

Participants took part in the subliminal priming test and a later assessment of awareness in a soundproof, dimly lit room. During the subliminal priming phase, each of the 60 trials started with a fixation point for 500 ms, immediately followed by a prime face for 12 ms and a scrambled mask for 500 ms. The mask in a given trial was created using the same face as for the prime in that trial. This prime-mask presentation was repeated three times before a target face was presented. Participants were asked to judge the facial expression (fearful or neutral) of the target face as quickly and accurately as possible (Figure 4). The target face disappeared either after participants responded, or after 2 s. All faces were pseudorandomly presented so that no more than three faces with the same valence were presented consecutively. The button press was counterbalanced across participants. Participants had a practice before the formal test.

During the post assessment phase, participants were first asked whether they saw anything between the fixation and the scrambled mask. They then completed a detection task to assess their objective awareness to the prime faces. The procedure was the same as the procedure for the subliminal priming phase. Participants were also assessed by the STAI before and after the experiment.

#### *Data Analysis*

The data on awareness detection were analyzed according to the signal detection theory. The measures of d′ and area under the ROC curve (A′) were calculated to characterize each participant's visual awareness [23]. Participants were considered as being aware of the prime when they claimed that they saw masked faces (subjective) or when their d′ or A′ values were significantly different from the chance level (objective) [50]. The significant *p* value was set as .05 (two tailed).

Accuracy and RTs of the affective priming task were recorded for each participant. Data from two participants were excluded from analysis due to their slower RTs (out of 2.5 SD of the mean). Among the remaining 59 participants, 18 participants were aware of the primes (7 as subjective and 11 as objective). The repeated measures ANOVA, Prime×Target×group, and Condition (congruent vs. incongruent)×group, were adopted. The priming effect was defined as the difference between congruent and incongruent conditions. Correspondingly, the priming effect for fearful faces was the difference between fear–fear and neutral–fear faces, and priming effect for neutral faces was the difference between neutral–neutral and fear–neutral faces. To match sample sizes between the two groups, the number of unaware participants was reduced to the similar number of aware participants (20 unaware, and 18 aware) based on their order to be enrolled in the experiment. The results of matched sample-size groups were similar to those of the complete groups, and were reported in detail. In addition, because participants responded to fearful faces more quickly than to neutral faces (534 ms vs. 556 ms), facilitated RTs in each condition (i.e., vs. scramble-fear or scramble-neutral condition) for each participant were calculated and analyzed. The results were similar to those of original data, thus only the results of original data were reported. SPSS was used for the analyses (*p*<.05, two tailed).

### Experiment 2

#### *Participants*

Seventy-four young (22±2.25 years old, 32 male) right-handed, healthy native Chinese-speaking students at Peking University took part in this experiment. They had normal or corrected-to-normal vision, and no history of neurological trauma or psychiatric disorders. All participants were paid for their participation and gave written informed consent in accordance with the procedures and protocols approved by the Human Participants Review Committee of Peking University.

#### *Materials and Procedure*

The materials were the same as those used in Experiment 1. For the experimental procedure, instead of the affective priming paradigm used in Experiment 1, a long-term priming paradigm was used, which consisted of four phases: unconscious encoding/study of faces, a subtraction task, a subsequent priming test, and post awareness assessments.

During the unconscious encoding/study phase (Figure 4), each of the 40 masked faces was presented 12 ms for six times. Then, to engage the participants' attention to focus on the center of the screen, a horizontal or vertical bar was presented, and participants were asked to judge its orientation as quickly and accurately as possible within 2 s. The priming test was performed after a delay of 3 min, during which a serial three-subtraction task was performed. During the test phase, each of the 40 prime faces and 20 new faces (10 fearful and 10 neutral) was presented on the center of the screen for 2 s. Of the 40 prime faces, half had the same facial expressions as those used in the subliminal encoding (congruent condition), and the other half had a new expression (incongruent condition). Participants were asked to decide whether the face was fearful or neutral as quickly and accurately as possible. The order of the faces was pseudorandomized so that no more than three faces with the same affective valence were presented consecutively. The button press was counterbalanced across participants. Participants had a practice before the formal test. The post awareness assessments were the same as that in Experiment 1.

#### *Data Analysis*

Data from three participants were excluded from analysis, one of them not following the instruction, and two having slower reaction times (out of 2.5 SD of mean). Among the remaining 71 participants, 23 participants were aware of the primes. To match sample sizes between the two groups, the number of unaware participants was reduced to the similar number of aware participants (24 unaware, and 23 aware) based on their order to be enrolled in the experiment. The data analysis was the same as that used in Experiment 1. The results of matched sample-size groups were similar to those of the complete groups, and were reported in detail.
